# Combination of strong anion exchange liquid chromatography with microchip capillary electrophoresis sodium dodecyl sulfate for rapid two-dimensional separations of complex protein mixtures

**DOI:** 10.1007/s00216-021-03797-4

**Published:** 2021-12-06

**Authors:** Holger Zagst, Christin Elgert, Sönke Behrends, Hermann Wätzig

**Affiliations:** 1 Technische Universität Braunschweig, Institute of Medicinal and Pharmaceutical Chemistry, Beethovenstraße 55, 38106 Braunschweig, Germany; 2 Technische Universität Braunschweig, Institute of Pharmacology, Toxicology and Clinical Pharmacy, Mendelssohnstraße 1, 38106 Braunschweig, Germany

**Keywords:** Two-dimensional separation, Capillary electrophoresis sodium dodecyl sulfate, Anion exchange high-performance liquid chromatography, Protein analysis

## Abstract

Two-dimensional separations provide a simple way to increase the resolution and peak capacity of complex protein separations. The feasibility of a recently developed instrumental approach for two-dimensional separations of proteins was evaluated. The approach is based on the general principle of two-dimensional gel electrophoresis. In the first dimension, semi-preparative strong anion exchange high-performance liquid chromatography is utilized and fractions are collected by means of a fraction collector. They are subsequently analyzed in the second dimension with microchip capillary electrophoresis sodium dodecyl sulfate. Microchip capillary electrophoresis provides the necessary speed (approximately 1 min/fraction) for short analysis. In this study, three different samples were investigated. Different constructs of soluble guanylyl cyclase were expressed in Sf9-cells using the baculovirus expression system. Cell lysates were analyzed and the resulting separations were compared. In our experimental setup, the soluble guanylyl cyclase was identified among hundreds of other proteins in these cell lysates, indicating its potential for screening, process control, or analysis. The results were validated by immunoblotting. Samples from Chinese hamster ovary cell culture before and after a purification step were investigated and approximately 9% less impurities could be observed. The separation patterns obtained for human plasma are closely similar to patterns obtained with two-dimensional gel electrophoresis and a total of 218 peaks could be observed. Overall, the approach was well applicable to all samples and, based on these results, further directions for improvements were identified.

**Graphical abstract:**

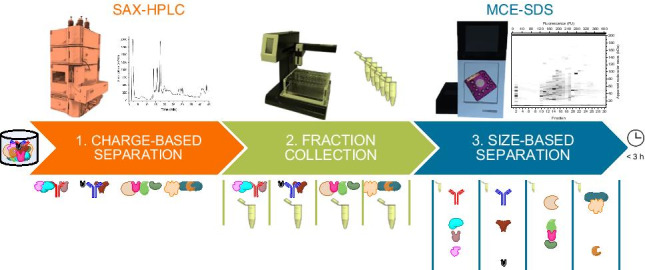
.

**Supplementary Information:**

The online version contains supplementary material available at 10.1007/s00216-021-03797-4.

## Introduction

The use of multi-dimensional separations for complex samples is a proven and tested strategy to facilitate an improved separation and easier analysis of complex samples, which are often insufficiently resolved using only one separation technique [1]. In the last century, two-dimensional gel electrophoresis (2-DE) emerged as one of the most successful technique for two-dimensional separations of complex protein mixtures [2].

With the advancement of analytical instruments, 2-DE has been replaced in many cases by automated instrumental approaches. Commonly employed strategies include high-performance liquid chromatography (HPLC) coupled to ( ×) HPLC, capillary electrophoresis (CE) × CE, and HPLC × CE approaches [3–8]. As pointed out by Ranjbar et al. [3], the popularity of HPLC × CE is much lower than HPLC × HPLC, despite the high orthogonality that can be achieved [3, 4, 6]. Such combinations provide a unique selectivity and may offer an alternative to other approaches. Even though, both capillary electrophoresis sodium dodecyl sulfate (CE-SDS) and ion exchange chromatography (IEX) are frequently employed for the characterization of proteins [9–12], the coupling of both techniques has not received much attention [13].

Within our group, an HPLC × CE approach for the separation of protein mixtures has been recently developed [14, 15]. It is based on the general principle of 2-DE, that is, a charge-based separation in the first dimension followed by a size-based separation.

The charge-based separation in the first dimension is realized by strong anion exchange (SAX) HPLC. The employed stationary phase is based on a poly(styrene–divinylbenzene) polymer. It is stable over a wide pH and pressure range and compatible with many commonly found additives and buffer excipients. Furthermore, it has a high loading capacity [16, 17]. An RP-1 guard column protects the SAX column from lipophilic contaminants. Sample preparation is minimal and is usually limited to dilution and filtration. For a comprehensive offline coupling, the eluate is automatically collected in individual fractions by a fraction collector. The resulting fractions are subsequently analyzed by microchip capillary electrophoresis sodium dodecyl sulfate (MCE-SDS). The MCE-SDS system is compatible with common buffer additives used in IEX [18]. Another advantage of the chip-based assay is time expenditure. For each run in the first dimension, a manifold of runs is required in the second dimension. Therefore, the second dimension is often the speed-limiting step during analysis. Regular CE-SDS runs take about 15 to 35 min, which is in the order of magnitude of a usual HPLC run. Therefore, several runs would represent a significant prolongation of an analysis. One possibility to circumvent this problem is the use of multiplexed capillary arrays [13]. In the presented approach, the chip-based separation is completed within 1 min, effectively eliminating this issue.

Our approach is intended as a platform approach, applicable to a wide range of samples with minor method adaptions. In this study, three different samples were investigated.

A commonly used production system for recombinant proteins are *Spodoptera frugiperda* (Sf9) cells [19]. In our case, the Sf9 cells were used for the production of soluble guanylyl cyclase (sGC), a heterodimeric heme protein which is the main receptor for nitric oxide [20]. Cell lysates of transfected and untransfected cells were analyzed. The applicability of our approach for biotechnological process control or analysis was evaluated. The identification of the sGC and the general practicability were the main concern.

The supernatant obtained from a Chinese hamster ovary (CHO) cell culture sample and its intermediate after a protein A purification step were subjected to analysis by our approach. The cell culture produced an immunoglobulin G1 (IgG) monoclonal antibody (mAb), which was secreted into the surrounding medium. Besides the product, several other proteins, commonly referred to as host cell proteins (HCP), are secreted by the cells. The HCP amount is usually regarded as critical quality attribute and monitored closely. Its minimization is an important objective during downstream processing [21–24]. Several analytical methods are used for HCP characterization, each with individual strengths and weaknesses [21, 25, 26]. Our approach might serve as an alternative analytical technique for the comparison or optimization of purification steps or as an orthogonal technique for the HCP determination.

Human plasma is a well-controlled product with a consistent composition [27–30]. Its proteome is well characterized and many proteins serve as biomarkers in diagnostic applications [30–33]. The proteins, whose individual concentration can differ over several orders of magnitude [31], are embedded in a complex matrix consisting of lipids, ions, carbohydrates, and more [29, 30]. Therefore, human plasma represents an ideal model to compare various approaches.

The goal of this study is to demonstrate the feasibility and applicability of the approach.

## Material and methods

### General

Bovine serum albumin (BSA; Fraction V (pH 7.0) for Western blotting), dithiothreitol (DTT; BioChemica grade), ethylenediaminetetraacetic acid (EDTA; molecular biology grade), glycerol (molecular biology grade), and sodium chloride (NaCl; for analysis) were obtained from PanReac AppliChem GmbH (Darmstadt, Germany). Sodium hydroxide (NaOH; EMSURE grade) and Amicon® Ultra-0.5 Ultracel-10 K centrifugal filter devices were purchased from Merck (Darmstadt, Germany). Sodium chloride (HPLC grade) and 2-amino-2-(hydroxymethyl)propane-1,3-diol (TRIS; electrophoresis grade) were obtained from VWR International GmbH (Darmstadt, Germany) and exclusively used for the HPLC experiments. Hydrochloric acid (HCl, p.a. grade), albumin from human serum (≥ 97%, HSA), albumin from chicken egg white (≥ 98%, ovalbumin), bovine serum albumin (≥ 96%, BSA), and β-lactoglobulin from bovine milk (≥ 85%) were acquired from Sigma-Aldrich (Steinheim, Germany). Matuzumab was received as a gift from Merck KGaA (Darmstadt, Germany). Syringe filters (polyvinylidene fluoride, 0.22 µm) and β-mercaptoethanol (β-ME, p.a. grade) were obtained from Carl Roth (Karlsruhe, Germany). Ultrapure water (conductivity 0.055 µS/cm) was supplied by an arium® pro VF system from Sartorius (Goettingen, Germany). Nylon membrane filters (0.2 µm pore size, 47 mm diameter) were supplied by GE Healthcare (Buckinghamshire, UK). The centrifuges 5417C and 5430 were purchased from Eppendorf (Hamburg, Germany). The pH measurements were done with a FiveEasy™ FE20 pH-meter and either an LE438 pH electrode (buffers) or an InLab Micro electrode (samples), all from Mettler Toledo (Gießen, Germany). All other chemicals were obtained from Sigma-Aldrich (Steinheim, Germany) in the highest grade of purity. Figures were plotted in OriginPro 2021 (9.8.0.200) from OriginLab Cooperation (Northampton, MA, USA). Calculations were performed using Microsoft® Excel® 2019 (v. 1809) from Microsoft Corporation (Redmond, WA, USA).

### HPLC system and fraction collector

The HPLC system (dwell volume 7.6 mL) consisted of a VWR Hitachi L-2130 quarternary pump, an L-2200 autosampler fitted with a 5.0 mL syringe kit, an L-2450 diode-array detector (all VWR International GmbH, Darmstadt, Germany), and a Techlab T1 column oven (Techlab, Braunschweig, Germany). The HPLC was controlled by EZChrom Elite (3.3.2 SP2, VWR International GmbH) and it was further used for data handling and integration. Directly coupled to the system was a Foxy R1 fraction collector (Knauer, Berlin, Germany), whose control was integrated into the HPLC software. The stationary phase was a semi-preparative PL-SAX 1000 Å, 8 µm, 50 × 7.5 mm column (Agilent Technologies, Waldbronn, Germany) protected by a 4 × 3.0 mm RP-1 guard column (Phenomenex, Aschaffenburg, Germany).

Mobile phase A (MPA) consisted of 20 mM TRIS pH 8.5, mobile phase B (MPB) consisted of 20 mM TRIS and 0.75 M NaCl pH 8.5, and mobile phase C (MPC) was composed of 20 mM TRIS and 1.5 M NaCl pH 8.5. The pH was adjusted to ± 0.05 units with 6 M HCl. All mobile phases were filtered through nylon membrane filters prior to use. If not otherwise indicated, the following method parameters were used. The column oven was thermostated at 30 °C, the flow rate was set to 1.3 mL/min, and UV absorption at 200 nm, 214 nm, and 280 nm (4 nm bandwidth, 2.5 Hz) was recorded. The injection volume is indicated at the respective samples. The following gradient was used for elution: from 0.0 to 5.0 min 100% MPA, followed by a linear gradient from 0% at 5.0 min to 100% MPB at 45.0 min. From 45.1 to 55.0 min 100% MPC and from 55.1 to 70.0 min 100% MPA was pumped through the system.

#### MCE-SDS

A LabChip® GX II Touch™ HT instrument, controlled with LabChip® GX Touch™ software (v. 1.7.819.0), was used with Protein Express LabChips and associated Protein Express kits (all PerkinElmer, Waltham, MA, USA). The HT Protein Express 200 high sensitivity assay was used. The proprietary chips are made of fused silica with a footprint of 37.25 mm × 37.25 mm. A plastic case (49.6 mm × 49.6 mm footprint, height up to 14 mm) is glued to the top of the chip and provides eight cylindrical wells for reagents and waste. A fused silica capillary (20–50 µm diameter, 24 mm length) is attached to the bottom of the chip as a “sipper” [34]. The “sipper” is used to hydrodynamically aspirate approximately 100–150 nL of the sample. The sample is subsequently moved electrokinetically into the separation channel (length 14 mm, width 31 µm) [35, 36]. A TRIS-Tricine buffer containing a non-cross-linked, linear, high molecular mass poly(dimethylmethacrylate) polymer, sodium dodecyl sulfate (SDS), and a proprietary dye is used as a sieving matrix for the size-based separation in an entangled polymer network at 30 °C [36, 37]. Laser-induced fluorescence (excitation 635 nm, emission 700 nm) is utilized for detection [38]. Further information about the detailed mode of operation is given by Chow [36] and in the associated patent [37]. In the preceding publication by Bousse et al. [39], a field strength of 200–300 V/cm is employed for the separations. The data was evaluated with the LabChip® GX Reviewer software (v. 5.5.2312.0, PerkinElmer).

### Protein concentration

Total protein concentrations were determined by the method of Bradford [40] using BSA as standard and Roti-Quant® (Carl Roth, Karlsruhe, Germany) as dye. Absorption at 470 and 595 nm was recorded by a Sunrise™ Absorbance Reader controlled and evaluated by Magellan™ software (both Tecan Deutschland GmbH, Crailsheim, Germany).

### Sample preparation

#### Sf9 cytosol lysate

Sf9 cells were obtained from the DSMZ (German Collection of Microorganisms and Cell Culture, Braunschweig, Germany). Soluble guanylyl cyclase subunits were recombinantly expressed using the baculovirus/Sf9 cell system. Recombinant viruses were generated as described in the Bac-to-Bac® Baculovirus Expression System manual [41] (Invitrogen™, Thermo Fisher Scientific, Waltham, USA). Sf9 cells were cultivated in Sf-900™ II Serum Free Medium (Gibco® by life technologies™, Thermo Fisher Scientific, Waltham, USA) supplemented with 10% fetal calf serum and 1% penicillin/streptomycin. Spinner cultures were grown at 27 °C and 140 rpm on a shaking incubator. For experiments, Sf9 cell density was adjusted to 2 × 10^6^ cells/mL and a volume of 100 mL was co-infected with baculoviruses encoding the sGC subunits: N-terminally twin strep tagged (TST) α_1_ [42, 43] and β_1_ [44] subunits. Fusion protein β_1_YFPα_1_ was cloned as previously described in [45]. Conjoined sGC β_1_α_1_ fusion construct was cloned as described in [46].

After 72 h of incubation, Sf9 cells were harvested by centrifugation (3020 × *g* at 4 °C for 2 min). The cell pellet was resuspended in lysis buffer (50 mM triethanolamine/HCl, 10 mM DTT, 1 mM EDTA, pH 7.4) containing complete™ protease inhibitor cocktail (Roche, Mannheim, Germany) and homogenized by ultrasound sonication. The samples were cleared by centrifugation (21,000 × *g* at 4 °C for 30 min). The cell lysates were diluted with MPA (1 + 1) and filtered through syringe filters. Injection volume was adjusted to the determined protein content so the injected protein amount remained comparable.

### Samples from CHO cell culture containing an IgG antibody

Cell culture supernatant and protein A purified IgG antibody, derived from a fed batch of a ready-to-use biosimilar CHO cell line using First CHOice® medium and feeds, were kindly provided by UGA Biopharma GmbH (Hennigsdorf, Germany). The frozen (− 20 °C) samples were thawed, vortexed (15 s), diluted with MPA (1 + 1), and filtered through syringe filters.

### Fresh-frozen human plasma

Anonymized leukocyte-depleted fresh-frozen human plasma was obtained from Städtisches Klinikum Braunschweig gGmbH and stored at − 32 °C. It was thawed in a tempered water-bath at 37 °C. The plasma was aliquoted and the aliquots were stored at − 32 °C. For use, aliquots were thawed as previously described and diluted with MPA (1 + 1), vortexed for 15 s, and filtered through syringe filters. Adjustments of the pH were done with 1% NaOH before filtration.

### Fraction collection and preparation for MCE-SDS

If not otherwise indicated, in each HPLC run, 30 fractions evenly spread over 45 min were collected (equals to 1.5 min or 1.95 mL per fraction). If the fractions were not processed immediately after collection, they were stored at − 20 °C and thawed for measurement. All fractions were vortexed 3 × 5 s and centrifuged at 10,000 × *g* for 3 min. Then, samples were prepared according to the manufacturer’s protocol [18]. Briefly, to 7 µL of sample buffer containing 3.4% (V/V) β-ME, 5 µL of each fraction were added in a 96-well plate. For non-reduced conditions, the sample buffer was used without the addition of β-ME. The plate was sealed and heated to 100 °C for 5 min. After cooling to room temperature, the plate was centrifuged at 1250 × *g* for 5 min; then, 32 µL water were added and mixed thoroughly. The plate was centrifuged again at 1250 × *g* for 5 min; then, the samples were measured with the LabChip®.

### Sodium dodecyl sulfate polyacrylamide gel electrophoresis (SDS-PAGE) and immunoblot analysis

For desalting, 500 µL from each sample’s 21^st^ fraction were transferred to an Amicon® filter device. Each capped device was centrifuged at 14,000 × *g* for 5 min. Then, 250 µL of MPA were added. Centrifugation at 14,000 × *g* for 5 min followed. This was repeated three more times. Finally, MPA was added to an approximate volume of 250 µL, the filter device inverted, and the desalted concentrate recovered by centrifugation for 2 min at 2060 × *g*.

SDS sample buffer (1% SDS, 50 mM TRIS/HCl, 100 mM DTT, 30% glycerol, pH 7.5) was added to each sample (raw lysates and desalted fractions) in a 1:1 ratio. Samples were vortexed for homogenization and heated to 99 °C for 3 min. After cooling, 80 µg of total protein was separated on a 10% SDS polyacrylamide electrophoresis gel. SDS-PAGEs were performed on the Mini-PROTEAN® Tetra System by Bio-Rad in a TRIS/glycine/SDS running buffer (25 mM TRIS, 192 mM glycine, 0.1% SDS, pH 8.3) and an applied voltage of 80 V for 4 h. The apparent molecular masses were determined by using PageRuler™ Unstained Protein Ladder and PageRuler™ Prestained Protein Ladder (both from ThermoFisher Scientific, Waltham, USA). After completion of the electrophoresis, the gels were washed with deionized water and proteins were transferred to nitrocellulose membranes using the Semi-Dry electro blotter Sedec™ M (Peqlab Biotechnology, Erlangen) with transfer buffer (25 mM TRIS, 192 mM glycine, 0.02% SDS, pH 8.3). Ponceau S staining, blocking of non-specific binding sites, and antibody incubation were performed as described in [45]. The following primary antibodies were used for detection: α_1_ (1:5000; Sigma-Aldrich G4280, Steinheim, Germany) and β_1_ (1:2000; Sigma-Aldrich G4405, Steinheim, Germany). Anti-rabbit IgG horseradish peroxidase–linked antibody (1:2000; Cell-Signaling Technologies 7074, Danvers, MA, USA) was used as secondary antibody for detection.

## Results and discussion

### Sf9 cytosol lysate

#### Two-dimensional separations

**Fig. 1:**
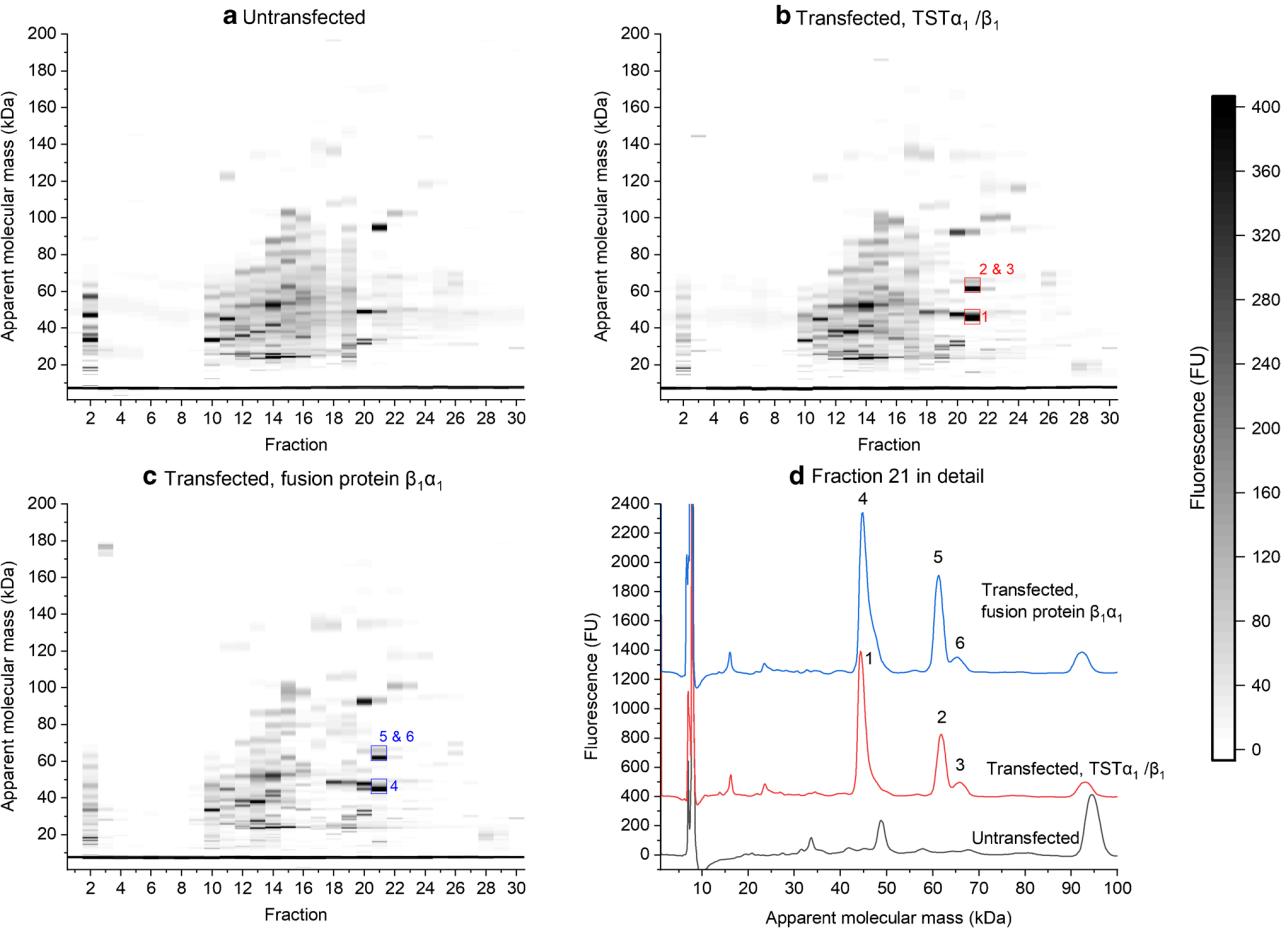
Two-dimensional separations (reduced conditions) of Sf9 cytosol lysate from **a** untransfected cells, 750 µl injected; **b** transfected cells expressing the sGC subunits TSTα_1_ and β_1_, 1500 µL injected; **c** transfected cells, expressing sGC fusion protein β_1_α_1_, 1423 µL injected; **d** shows the individual electropherograms of the 21^st^ fraction of each separation, bottom (black) untransfected sample, middle (red) transfected sample TSTα_1_ / β_1_ (offset 400 FU), and top (blue) transfected fusion protein sample (offset 1250 FU); peaks 1–6 are attributed to the sGC

The resulting two-dimensional separations of the three samples are shown in Fig. 1. The total protein concentrations of the samples before dilution were 49.6 mg/mL (untransfected), 24.8 mg/mL (transfected with TSTα_1_/β_1_), and 26.2 mg/mL (transfected with fusion protein β_1_α_1_). The Sf9 cytosol lysates show very similar peak patterns across all three samples. Most of the peaks were found in fraction 2 and 10 to 22. The peaks are primarily found within the 10 – 100 kDa range. The ever-present peak at approximately 7 kDa is a system peak originating from SDS micelles. Most peaks were present in all samples, forming a characteristic peak pattern. There are some variations in their intensity, which is in accordance with the expectations, as the LabChip® assay has some variety [18]. Additionally, cells, as a living organism, have an inherent biological variety. The major difference between the samples was observed in fraction 21. Here, both transfected samples, but not the untransfected sample, clearly displayed a peak at 61 kDa (Peak 2 in Fig. 1b/d) with a shoulder peak at 65 kDa (Peak 3 in Fig. 1b/d). This difference corresponds to a differently shaped peak at approximately 30 min in the HPLC chromatogram for both transfected samples (SI Fig. 1). It is presumed that this peak pair in the electropherogram can be attributed to the α_1_ and β_1_ subunit of the sGC with a molecular mass of approximately 77 kDa and 70 kDa, respectively [47]. The assay’s sizing accuracy is defined with ± 20% [18] and no major differences in this size range were found in other fractions, further reassuring this hypothesis. An additional peak at 44 kDa (Peak 1 in Fig. 1b/d) is also noticeable. Surprisingly, the sample containing the fusion protein also showed peaks at 61 and 65 kDa (Peaks 5 and 6 in Fig. 1c/d), instead of one expected peak at around 150 kDa. The additional peak at 44 kDa (Peak 4 in Fig. 1c/d) was also present. This indicates that the protein might be instable under the given conditions. The experiment was repeated to verify the results, albeit including cells expressing a slightly modified fusion construct, containing an additional tag (β_1_YFPα_1_ [45]). As in the preceding experiment, a peak around 46 kDa and peak pair at 61 kDa and 65 kDa could be observed in the 21^st^ fraction of each transfected samples, but not in the untransfected sample’s fraction 21 (SI Fig. 2).

### Validation of peak identity

**Fig. 2:**
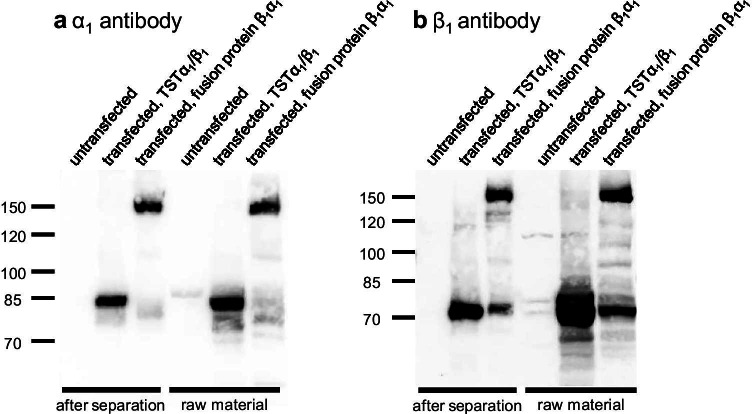
Cutouts of the immunoblots of the 21^st^ fraction of each separation depicted in Fig. 1 (“after separation”) and of the corresponding raw Sf9 cytosol lysates (“raw material”), with primary antibody for α_1_ subunit (**a**) and with primary antibody for β_1_ subunit (**b**); position of the ladder proteins and their nominal molecular mass are indicated on the left side of the images

Since these results still left some uncertainty about the peak identity, each sample’s fraction 21 as well as the raw cell lysates were separated by SDS-PAGE and subsequently analyzed by immunoblotting. The α_1_ and β_1_ subunits were found in all lysates from transfected cells and in every corresponding 21st fraction. All untransfected samples were devoid of these two proteins (Fig. 2). The fractions containing the fusion protein display bands for the β_1_ subunit not only around 150 kDa, but also slightly above 70 kDa. The corresponding samples with the raw cytosol display the same bands (Fig. 2b). Similar results were obtained for the α_1_ subunit; however, the samples containing the fusion protein only had a minor band at the expected position of the individual α_1_ subunit (Fig. 2a). Based on practical experience with the α_1_ subunit, it appears to be less stable than the β_1_ subunit. The observed instability of the fusion protein might be the result of protein degradation, including the separation into the respective subunits. Since the observed instability applies to all samples, raw material, and individual fractions, it is most likely not limited to our method, but its conditions seem to amplify the degradation, especially in the case of the fusion protein. Overall, these findings support the observations of the two-dimensional separations and offer a reasonable explanation for them. The results could be confirmed using the raw lysates and corresponding 21^st^ fraction from the repeated experiment containing the β_1_YFPα_1_ fusion protein (SI Fig. 3).

Commonly, the peak identity is confirmed by mass spectrometry (MS) [4, 48–50]. MCE-SDS and CE-SDS in general are considered as incompatible with MS due to the intrinsic properties of the employed gel matrix. Nevertheless, approaches to address this issue have been published [51–53], with the most recent one [51] showing promising results. These approaches are currently limited to regular CE-SDS and do not include chip-based formats. However, recent progresses in microchip-based CIEF-MS [49] raise hope that similar successes may be achieved with MCE-SDS.

During the SAX-HPLC run, the employed salt gradient for the elution introduces a large amount of nonvolatile ions, effectively eliminating the possibility of using MS. However, the individual fractions contain only a fractional amount of the initial total protein amount, reducing the complexity of the sample. By means of buffer exchange to a volatile, MS compatible buffer, the individual fractions could be analyzed with MS. Direct coupling of cation [50] and anion exchange chromatography [12] with MS has been reported. The employed strategies could also be applied for further developments of the first dimension if MS compatibility is desired.

### Samples from CHO cell culture containing an IgG antibody

**Fig. 3:**
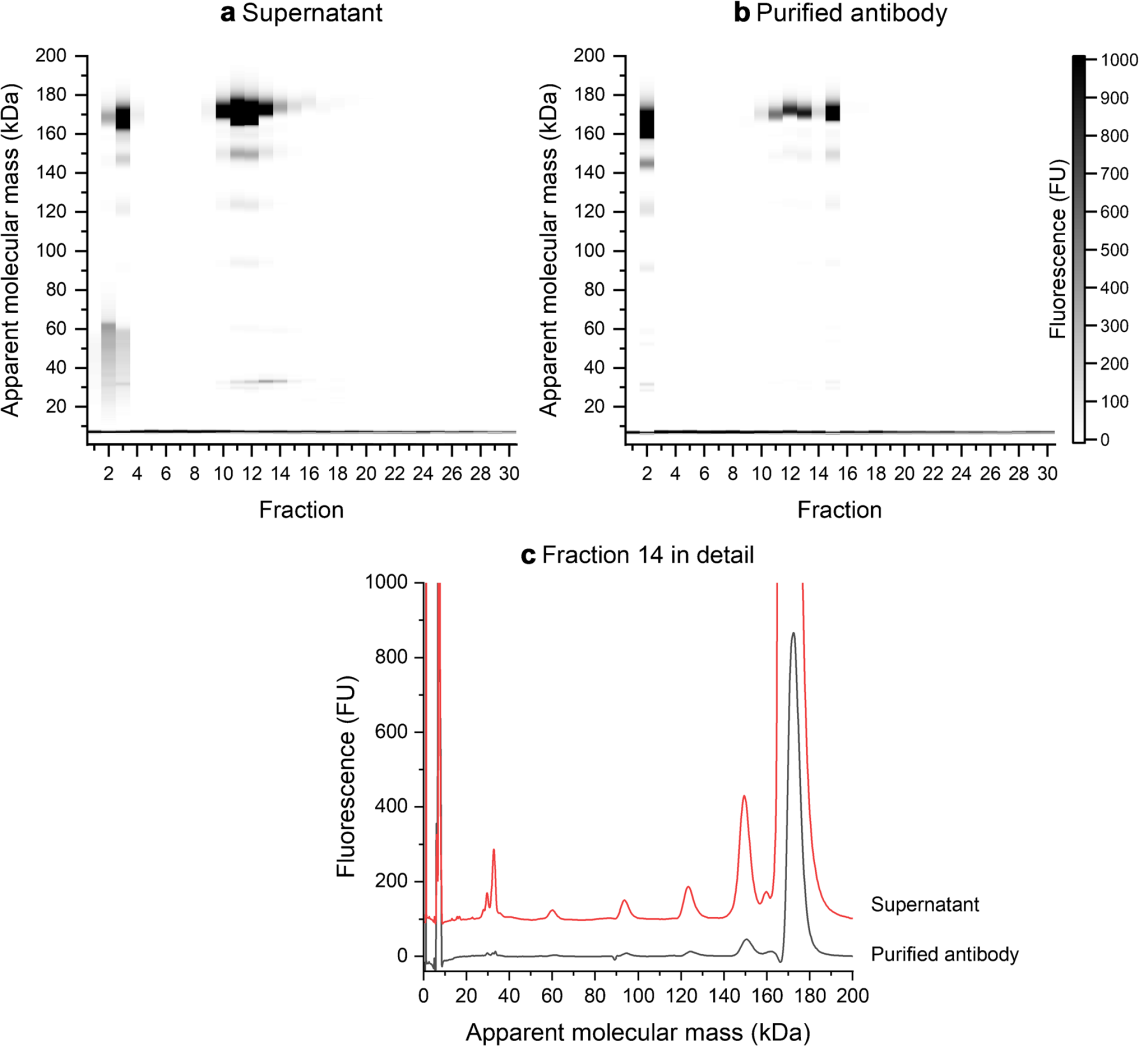
Two-dimensional separations (non-reduced conditions) of CHO cell culture supernatant before (**a**) and after protein A purification (purified antibody, **b**); injection volume 3000 µL for both; in **c**, two exemplary electropherograms of the 14^th^ fraction of each separation are depicted, the upper red trace from the supernatant and the lower, black trace from the purified mAb. The intensity of the impurity peaks (10–165 kDa) is greatly reduced in the black trace

The resulting separations are shown in Fig. 3. The peak at 7 kDa is the aforementioned system peak. Most protein is contained in fractions 2, 3, and 10–15. The peak around 175 kDa accounts for most of the protein. In this example, peaks were assigned based on their apparent molecular mass. All peaks with approximately 175 kDa were assigned as IgG, while the rest of the peaks was regarded as impurities. Non-reduced conditions were chosen, due to a possible overlap of the peaks from the IgG light chain and impurities around 30 kDa under reduced conditions. The majority of the impurities can be found in the same fractions as the IgG (2, 3, 10–15), effectively overlapping with the elution of the IgG antibody during the SAX-HPLC run. The effect of the protein A purification can be seen in Fig. 3b. The total percentage area of non-IgG peaks is reduced, from about 17 to 8%. The effect is also visible in the exemplary electropherograms in Fig. 3c. The total protein concentration of the supernatant and purified antibody solution was 4.5 mg/mL and 3.1 mg/mL, respectively. Due to that, the results should be seen with caution. The isoelectric point (pI) of many IgG mAbs is found in basic regions [54]. Improved retention and separation might be obtained using strong cation exchange instead of SAX and could be an alternative for similar samples in the future.

### Fresh-frozen human plasma

**Fig. 4:**
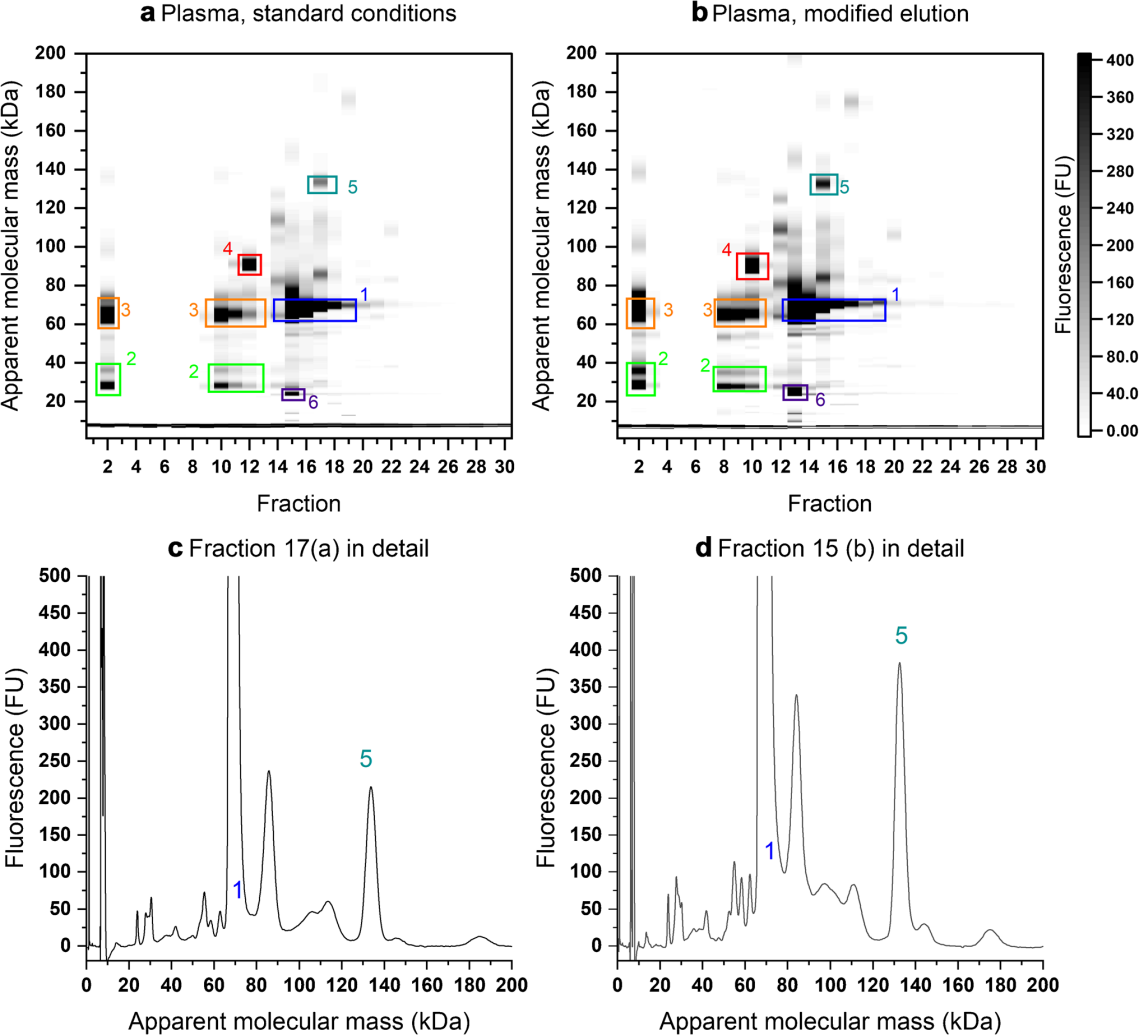
Two-dimensional separations (reduced conditions) of 500 µL human plasma under standard conditions (**a**) and with modified elution: flow rate 1 mL/min, linear gradient from 0 to 100% MPB over 40 min (**b**), the peaks are presumed to be albumin (1), Ig light chains (2), Ig heavy chains (3), serotransferrin (4), ceruloplasmin (5), and apolipoprotein A1 (6); detailed view of two electropherograms from fraction 17 of the separation in 4a (**c**) and from fraction 15 of the separation in 4b (**d**), peaks 1 and 5 are indicated in both traces

An exemplary separation of 500 µL human plasma is depicted in Fig. 4a. The protein concentration of the injected solution was 30.5 mg/mL. Most peaks were found in the 2^nd^ and 10^th^ to 19^th^ fraction. Only few peaks above 120 kDa are visible.

The human plasma proteome is well known [30, 31, 55] and classical 2-DE employs a comparable separation mechanism. A detailed depiction of a 2-DE separation of human plasma is provided in [56]. Further 2-DE separations may be found in several other publications [57, 58]. Generally, the elution order in our approach is the opposite as in 2-DE, from basic to acidic proteins. The proposed assignment of the peaks is based on their relative abundance, their apparent molecular mass, and their relative positions to each other. The most abundant protein is albumin [31] (calculated molecular mass based on amino acid sequence (cMM) 66 kDa [30]), which corresponds to peaks “1” (blue box) around 64 kDa in fractions 15 to 19. In fraction 2 and in 10 to 12, the peaks “2” (green box) around 24 and 27 kDa presumably are immunoglobulin light chains (cMM 23 kDa [30]) and peaks “3” (orange box) around 65 kDa (cMM 53–75 kDa [30]) the corresponding heavy chains. The peak “4” (red box) at 90 kDa in fraction 12 could be serotransferrin (cMM 75 kDa [30]). Both, immunoglobulins and serotransferrin, can be found at more basic pIs than albumin in 2-DE [56]. Furthermore, the peak “5” (petrol box) at 134 kDa in fraction 17 could be ceruloplasmin (cMM 120 kDa [30]) and the peak “6” (purple box) in fraction 15 around 24 kDa might be apolipoprotein A1 (cMM 28 kDa [30]). Overall, the pattern seems similar to those of the aforementioned 2-DE separations, if the assignment is correct. For a definitive statement, confirmation of peak identity would be necessary.

Several changes of the method were conducted and tested. The short isocratic hold time (5 min) at the beginning of the method was removed. The dwell volume of the used HPLC system is large (7.6 mL). Therefore, the gradient needs about 5.85 min at 1.3 mL/min (or 7.6 min at 1 mL/min) until it reaches the column inlet. This should be a sufficient isocratic hold time for a gradient method. As a result, the same separation in a shorter timeframe (40 min) was obtained. The number of fractions remained constant, effectively reducing the individual fraction’s volume and increasing the sensitivity. Second, the flow rate was reduced to 1 mL/min. This yielded a higher sensitivity without impairing the separation. The combined effect of both changes can be seen in Fig. 4b. In comparison to Fig. 4a, the separation remains unaffected but is shifted leftwards, two fractions earlier. Additionally, the intensity of the peaks increases. This is very well visible, but, due to the chosen gray-scale, only for the low abundant peaks. For example, the intensity of the peak “5” around 133 kDa in fraction 17 (standard conditions, Fig. 4c), respective 15 (modified conditions, Fig. 4d), increased from 215 to 382 FU. Nevertheless, it applies also to the high abundant peaks, e.g., the peak “1” around 67 kDa in fraction 17 (standard conditions) has an intensity of ≈ 3000 FU, while in fraction 15 (modified conditions) the intensity is at about 5700 FU. The effect is also visible in the chromatograms. The peak intensity is increased and the peaks are shifted to earlier retention times while preserving the original peak pattern (SI Fig. 4).

The pH value of the sample might influence the retention and therefore the separation. The sample’s pH was increased from 8.24 to 9.59 and 10.55. Higher pH led to an unchanged separation, with some decrease in peak intensity (data not shown). This might be due to precipitation. Overall, the separation is robust to a basic shift in the sample’s pH value.

Due to the limited protein capacity of the column, the high abundance of albumin creates a challenge for the detection of proteins with much lower concentrations. The proven and tested strategies used in 2-DE for the depletion of the most abundant proteins [59, 60] could also be applied here and should allow for more individual proteins to be detected.

### Performance

#### Repeatability

Three consecutive injections of 750 µL Sf9 cytosol lysate (53.4 mg/mL before dilution) were analyzed. The following method parameters differed from the described parameters: 1 + 1 dilution with lysis buffer, 25 fractions over 37.5 min were collected. A peak with a comparatively low intensity (signal to noise ratio (S/N) ≈ 5) and a peak with a normal intensity (S/N > 100) were investigated. The low intensity peak can be seen as a “worst-case scenario,” while the normal intensity peak represents a normal use case. The S/N was calculated according to the European Pharmacopoeia [61]. The electropherograms were integrated and all peaks within the assay’s specified range (10–200 kDa) included. Time corrected areas (corr. area) were obtained from the LabChip’s software and used for further calculation. The relative standard deviation (RSD) of the corr. area and of the percentage of the corr. area of the individual peak to the total corr. area of all peaks in the fraction (%area) was calculated. The RSDs for the low intensity peak were 15.5% (corr. area) and 21.9% (%area), while for the normal intensity peak they were 20.6% (corr. area) and 10.1% (%area). These values are in the order of magnitude of previously reported values for this particular LabChip® assay [62] and within the specifications given by the manufacturer (up to 30% RSD for quantitation) [18]. Improved values might be obtained by averaging the values from multiple MCE-SDS injections of the same sample at the expense of speed [62]. For this sample, injecting every fraction twice improved the RSD for the normal intensity peak to 17.7% (corr. area) and 9.73% (%area). The RSDs for the low intensity peak did not benefit from this, with values of 23.2% (corr. area) and 21.7% (%area). In this case, the low S/N has a major influence on precision, since the S/N needs to be > 100 for optimal precision [63]. Further improvements may be achieved with the ProteinEXact assay, which has tighter specification limits for quantification [64, 65].

### Expected sensitivity

The MCE-SDS assay’s linear concentration range starts at 5 ng/µL [18], and Kahle et al. [62] reported an S/N of 44 at 10 ng/µL for carbonic anhydrase. One fraction contains about 1950 µL eluate, division through the aforementioned 5 ng/µL leads to at least 9.75 µg of protein required per fraction. Assuming a recovery of 85%, at least 11.5 µg need to be injected $(\frac{9.75 \mu g}{85\%} =11.5 \mu g)$. The typical injection volume ranges from 100 to 3000 µL. Assuming 1000 µL are injected, the protein’s concentration should be 11.5 ng/µL or higher to be detectable.

Porebski et al. [66] report an influence of the salt concentration on peak areas during CE-SDS. Since the LabChip® moves the sample electrokinetically into the separation channel [35], an influence on the areas can also be reasonably expected here. This was confirmed by adding equal amounts of BSA to each fraction of a blind run. The peak area is negatively correlated with the salt concentration (SI Fig. 5). This should be continued to be investigated in conjunction with further method changes. The previously described method changes represent a good starting point. Another possibility is the use of the Protein Pico Assay. Here, a sensitivity of approximately 10–50 pg/µL, which corresponds to a 100-fold improvement over the Protein Express Assay, can be expected [67]. The drawback is that a buffer exchange would be necessary. Other two-dimensional HPLC × CE approaches use evaporation and reconstitution [68]. In our case, this would unfavorably increase the salt concentration.

### Peak capacity

An easy and convenient way for the assessment of the peak capacity is simply counting peaks. Each MCE-SDS run’s electropherogram was integrated and all peaks with an apparent molecular mass ≥ 10 kDa were counted. The results from all 30 electropherograms were summed. For the most complex sample, namely Sf9 cytosol lysate, between 330 (sample Fig. 1a) and 419 peaks (sample Fig. 1b) were achieved. Human plasma (sample Fig. 4b) counted 218 peaks. One possibility to increase the peak capacity could be an increased amount of fractions. For HPLC × HPLC separations, the requirement was stated that ideally each peak in the first dimension should be sampled at least three to four times [69]. This concept has been refined in subsequent publications [70, 71], and moreover, it has been frequently cited. However, to be precise, this concept and derived concepts are only applicable for HPLC × HPLC separations. Since the theories about peak capacity, sampling frequency, etc. are not as well developed for CE based separations [72], it can only be assumed that a similar impact can be achieved here. Further refinements of the first dimension may provide an additional option to increase the peak capacity.

### Speed

The presented approach uses commercially available instruments, which can be set up and operated in any lab. This facilitates an easy implementation. The approach is fast and might be accelerated even more. The expected run time for one sample is approximately 3 h. The time is based on the following assumptions: 45 min HPLC preparation, 15 min sample preparation, 45 min HPLC separation, 10 min fraction handling, 30 min fraction preparation for MCE-SDS, 5 min chip preparation and cleaning, 30 min MCE-SDS runs. An online approach is not easily feasible due to the required heating during sample preparation for the 2^nd^ dimension. Several other possibilities arise to streamline the process. Running consecutive analyses and the second dimension in parallel with the first dimension of the next samples reduces analysis time to approximately 1.5 h per sample with the present setup. The initial publication describing the stationary phase points out the possibility of using high linear velocities [16]. In a preliminary experiment using human plasma, the flow rate was doubled from 1 to 2 mL/min. Consequently, the HPLC run was completed within 35 min. The resulting chromatogram and two-dimensional separation are shown in SI Fig. 6. This is another promising possibility to increase the separation speed and will be the scope of further investigations.

Currently, the fractions are collected in 2-mL microcentrifuge tubes. With the appropriate, commercially available equipment, fraction collection directly in 96-well plates is possible. The required reduced flow rate could be either obtained using a flow splitter or by miniaturization of the separation process. The collection into a 96-well plate would speed up the preparation process for the MCE-SDS analysis considerably. Another possibility is the automation of the remaining manual steps. Particularly pipetting could be sped up by automation and reduce hands-on time. If one is routinely analyzing the same sample and is only interested in particular proteins, an adaption of the gradient and reduction of the fraction amount could further increase the speed. This implies a switch from a comprehensive to a heart-cutting approach.

### Size and pI range

The size-based assay is suited for apparent molecular masses between 10 and 200 kDa. Below 10 kDa, system peaks interfere with potential sample peaks. In the different samples, proteins over the whole range were found. The Protein Low Molecular Weight Assay could offer an alternative for proteins within the 5–80 kDa range, but a buffer exchange is required for fractions containing more than 0.5 M NaCl [73].

Generally speaking, proteins need to possess a negative charge to be retained on the column. As a rule of thumb, this necessitates a pI below 7.5–6.5 [9], the lower the better the retention. Several individual proteins were analyzed by SAX chromatography at a concentration of 1 mg/mL in MPA. The resulting chromatograms are presented in SI Fig. 7. Matuzumab is a mAb with a pI of 8.3 (main species)–7.5 (SI Fig. 8). The peak’s maximum is at 14.7 min, which is shortly after the beginning of the salt gradient. The other proteins possess a lower pI, Ovalbumin 4.7–4.9 [74, 75], BSA 4.6–5 [76–78], HSA 4.7–5.7 [79–81], and β-Lactoglobulin 5.2 [82, 83]. Their retention times were 21.3 min (Ovalbumin), 24.3 min (BSA), 24.8 min (HSA), and 26.1 min (β-Lactoglobulin). The proteins identified in the human plasma sample are found between pI 4 and 8 in 2-DE [58]. Based on these results, the estimated covered pI range is from about 4 to 8. However, non-retained proteins are not lost, but simply found in the first fractions.

These considerations should also take into account that the net charge alone is not always sufficient for the explanation of the retention. The (unequal) surface charge distribution, the displacing salt, stationary phase, and protein–protein interactions may influence the retention and sometimes complicate general assumptions [84, 85].

The aforementioned use of a cation exchange column instead of an anion exchange column could expand the covered range through the altered selectivity. This might be relevant if the attention is on basic proteins and will be subject of further investigations.

## Conclusion

The presented approach was very well applicable to the three different examples, clearly demonstrating its feasibility. The IgG containing cell culture supernatant as well as the Sf9 cytosol lysate demonstrated the use of the approach for biotechnological process control or analysis. The latter one focused on a specific protein in a complex matrix and the former focused on the changes during a process. The protein sGC could be confidently identified. The results from the human plasma separation and their comparison to conventional 2-DE results revealed that the underlying comparable separation mechanism might lead to similar but not directly transferable results.

In all cases, quick two-dimensional separations were achieved. The potential for further acceleration of the separation process was highlighted. Method improvements, especially in the first dimension, should further improve this approach. Potential steps were outlined and briefly discussed. Using best-suited separation systems in the first dimension can also customize this approach for individual requirements and sample properties. The second dimension offers fewer opportunities for refinement.

With refined parameters, the determination of additional relevant method parameters, e.g., reproducibility, linearity, robustness, etc., is of interest. Further research with the objective of expanding the applications to e.g. impurity analysis, diagnostics, or process analytics will help to gain a more thorough understanding of the technique’s properties.

## Supplementary Information

Below is the link to the electronic supplementary material.

Supplementary file1 (PDF 624 kb)

## Data Availability

The datasets generated during and/or analyzed during the current study are available from the corresponding author on reasonable request.
