# Supplementary material for: Combination of strong anion exchange liquid chromatography with microchip capillary electrophoresis sodium dodecyl sulfate for rapid two-dimensional separations of complex protein mixtures
Source: Anal Bioanal Chem. 2021 Dec 6;414(4):1699–712. doi: 10.1007/s00216-021-03797-4 (PMC8761713; doi:10.1007/s00216-021-03797-4)
Supplement: Supplementary file 1 — Supplementary file1 (PDF 624 kb) [file 216_2021_3797_MOESM1_ESM.docx]

## Supporting Information


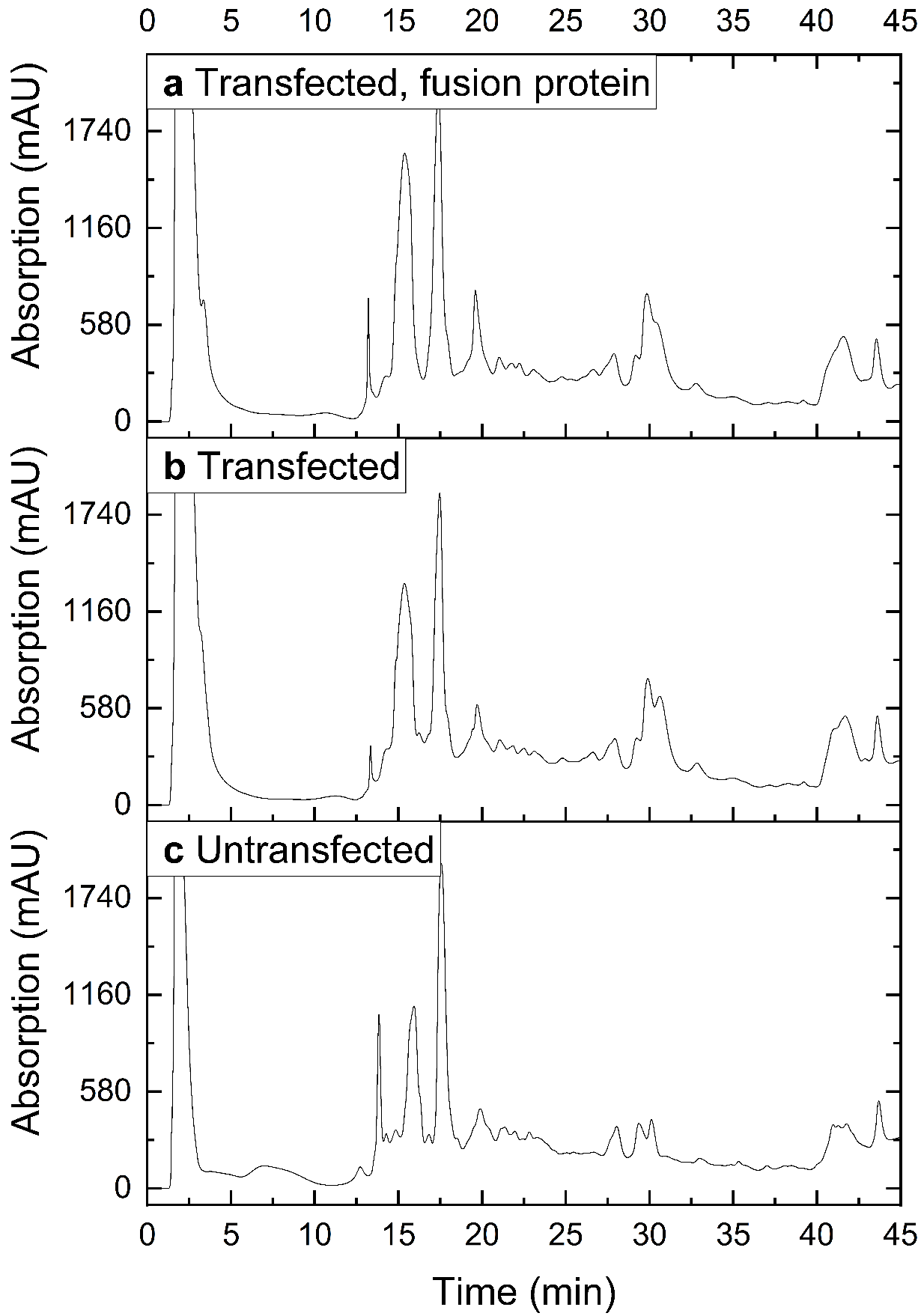


**SI Fig. 1** Three HPLC chromatograms (280 nm) of the separation of Sf9 cytosol lysates, corresponding to the two-dimensional plots Fig. 1a (untransfected sample, bottom, **c**), Fig. 1b (transfected sample expressing TST α_1_/ β_1_, middle, **b**) and Fig. 1c (transfected sample, expressing fusion protein β_1_α_1_, top, **a**). The altered peak shape around 30 min corresponds to the difference observed in fraction 21 (Fig. 1d).


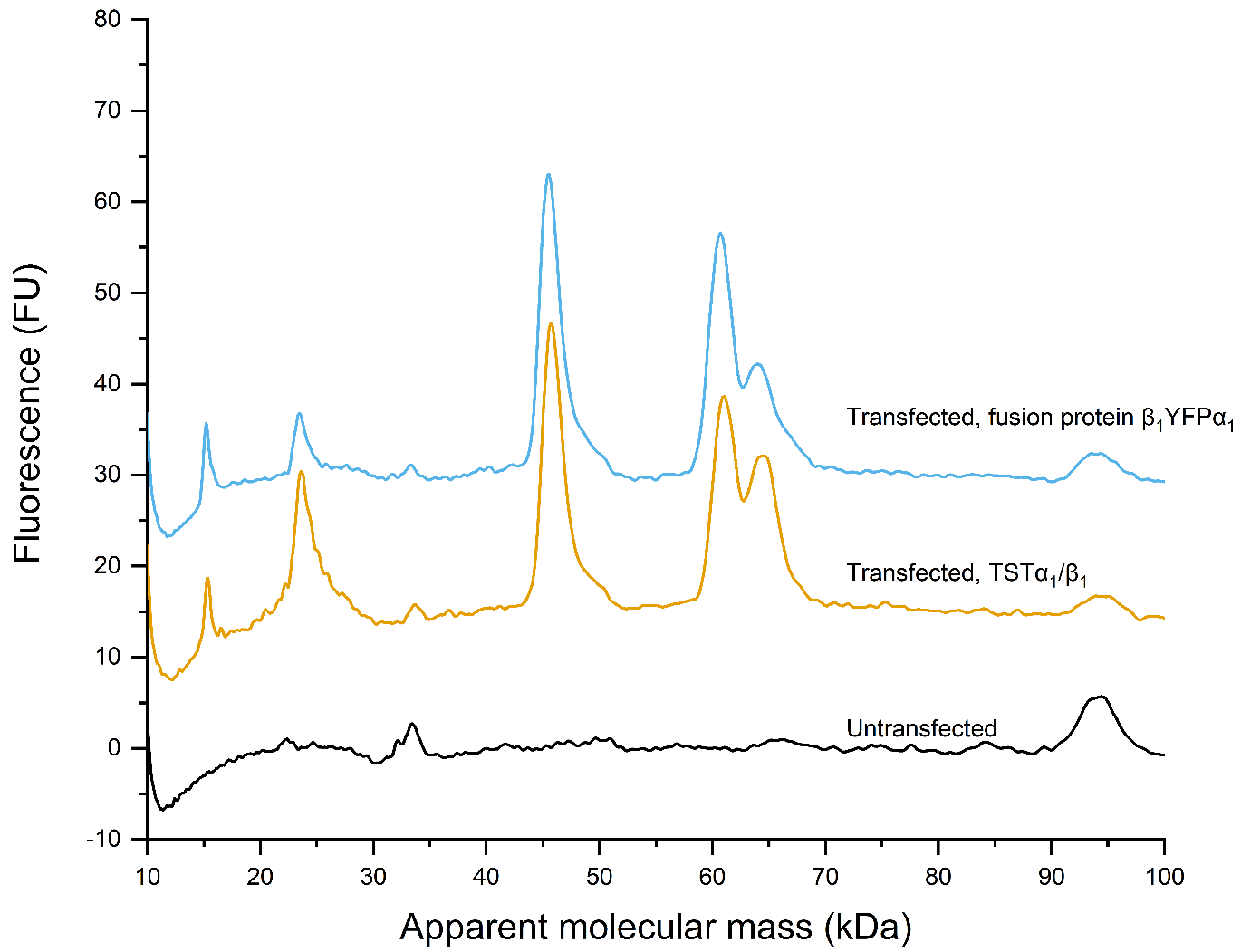


**SI Fig. 2**Individual electropherograms of the 21^st^ fraction of each separation from the repeated experiment; bottom (black) untransfected sample, middle(orange) transfected sample TSTα_1_ / β_1_, top (blue) transfected fusion protein (β_1_YFPα_1_) sample; each with 15 FU offset. The differences at 46, 61 and 65 kDa, observed in the previous experiment (Fig. 1d), are also clearly visible here.


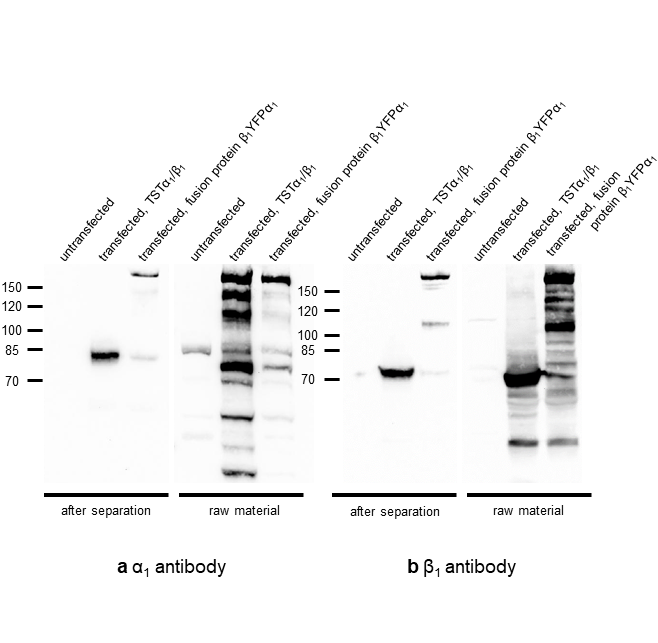


**SI Fig. 3** Cutouts of the immunoblots of the 21^st^ fraction of the repeated experiment depicted in SI Fig. 2 (“after separation”) and of the corresponding raw Sf9 cytosol lysates (“raw material”), with primary antibody for α_1_ subunit (**a**) and with primary antibody for β_1_ subunit (**b**); position of the ladder proteins and their nominal molecular mass are indicated on the left side of the images.


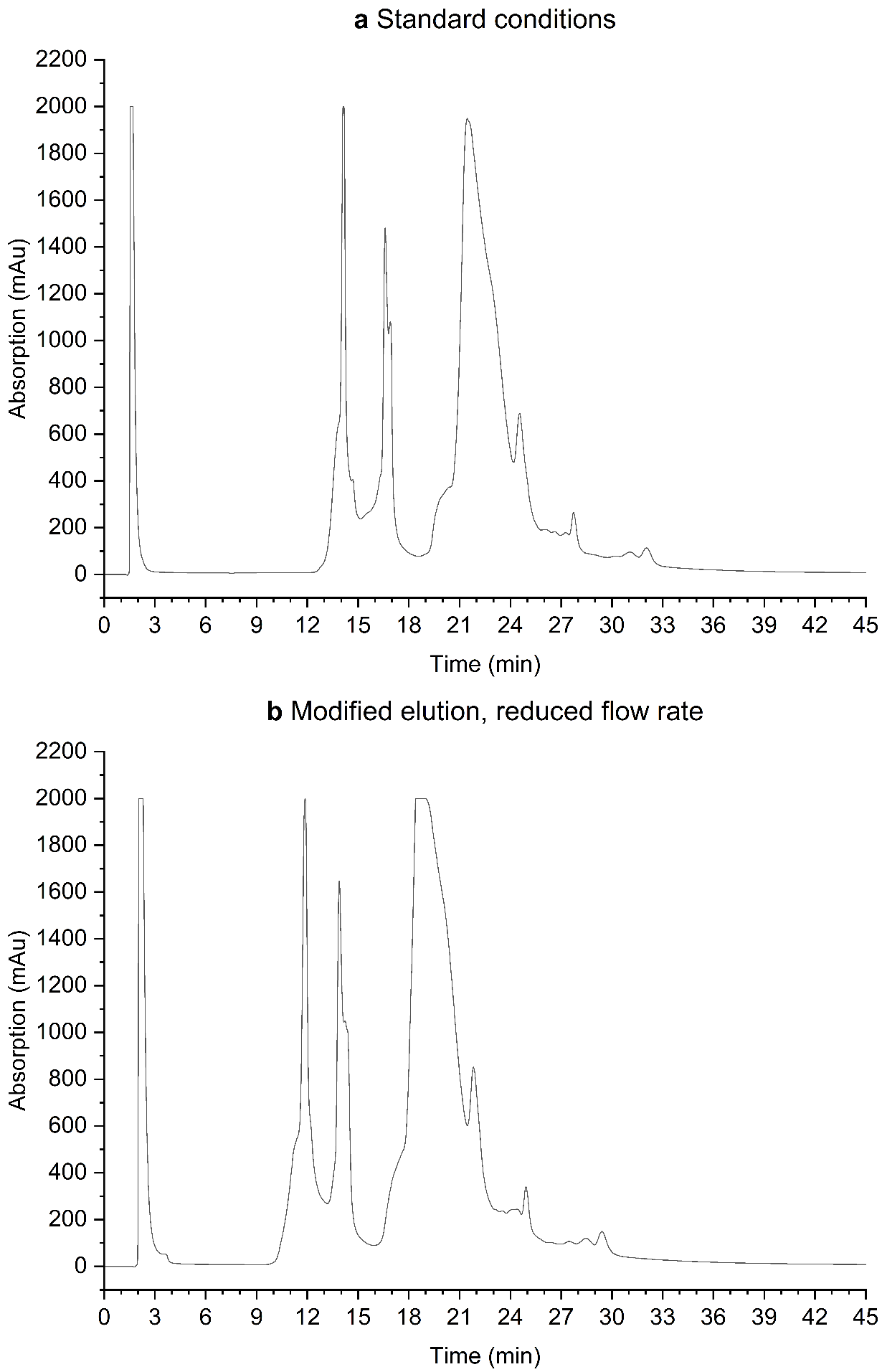


**SI Fig. 4** HPLC chromatograms (280 nm) of 500 µL human plasma using standard conditions (**a**) and a modified gradient (linear gradient from 0% to 100% MPB over 40 min) and a flow rate of 1 mL/min (**b**).


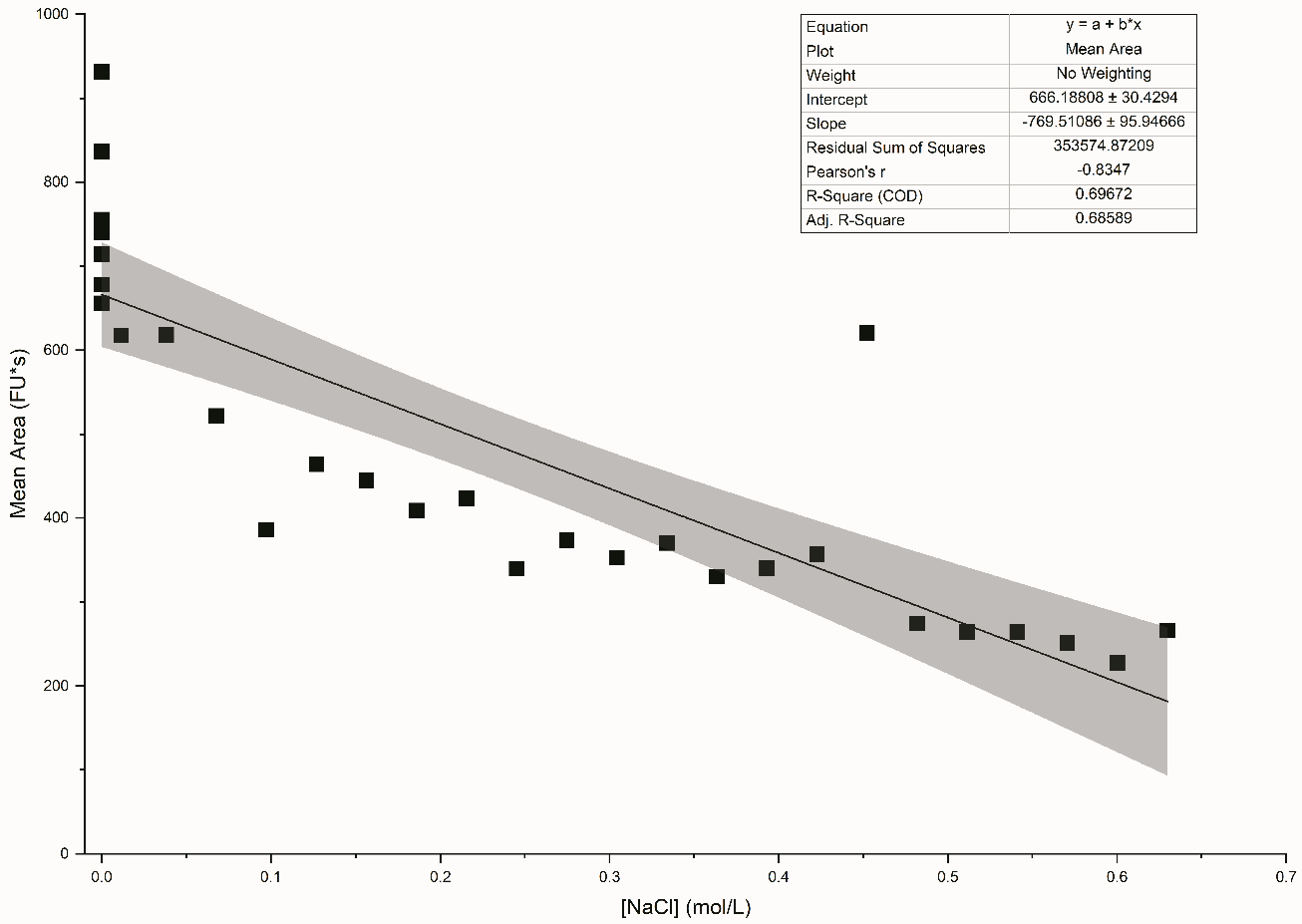


**SI Fig. 5** Relationship between mean corrected area and sodium chloride (NaCl) concentration of the fraction for bovine serum albumin. To each fraction of a blind run, the same amount of BSA was added to a final concentration of 0.5 mg/mL. The NaCl concentration was calculated based on the system parameters. Each point represents the mean of three LabChip measurements. Linear regression was performed. Grey area represents the 95% confidence interval of the regression function. The 95% confidence interval of the slope does not include 0, therefore a trend is assumed.


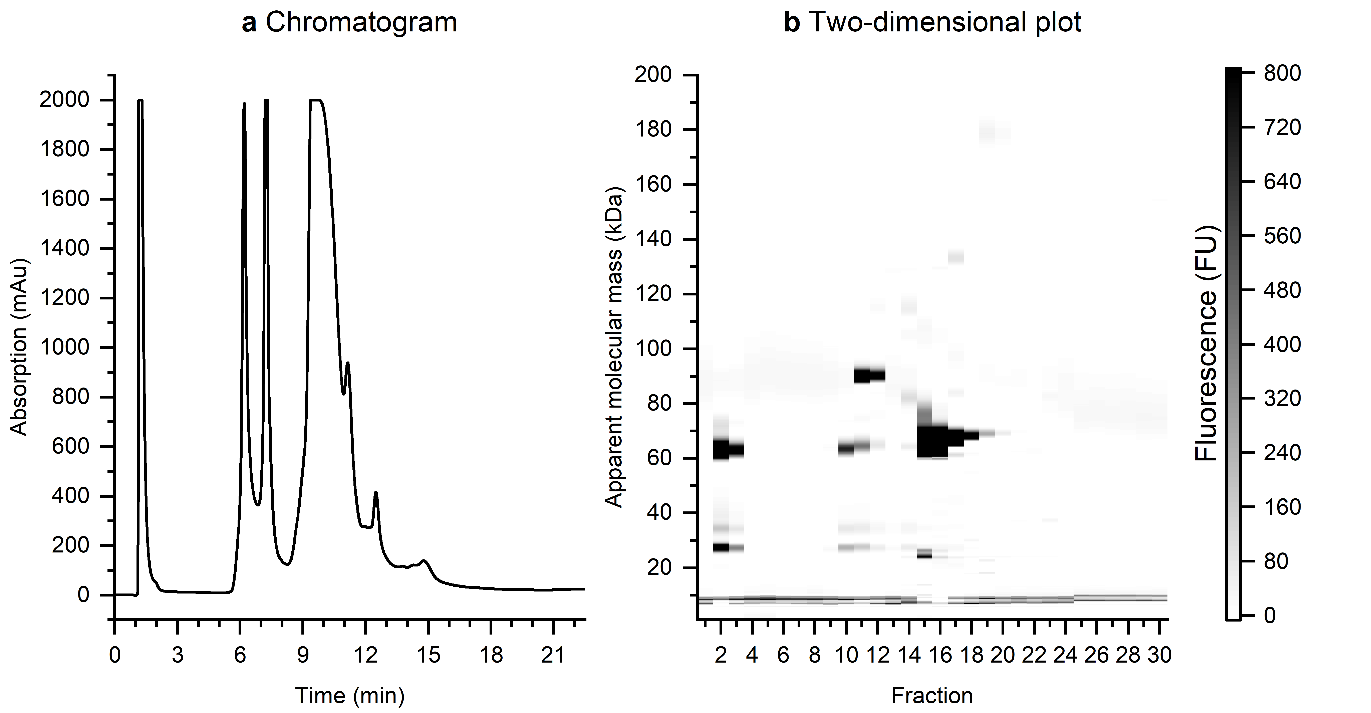


**SI Fig. 6** HPLC chromatogram (280 nm, **a**) and the obtained two-dimensional plot (**b**) of the separation of 500 µL human plasma diluted 1+1 with MPA, flow rate of 2 mL/min, linear gradient from 0% to 100% MPB over 20 min, 30 fractions equally distributed over 20 min were collected.


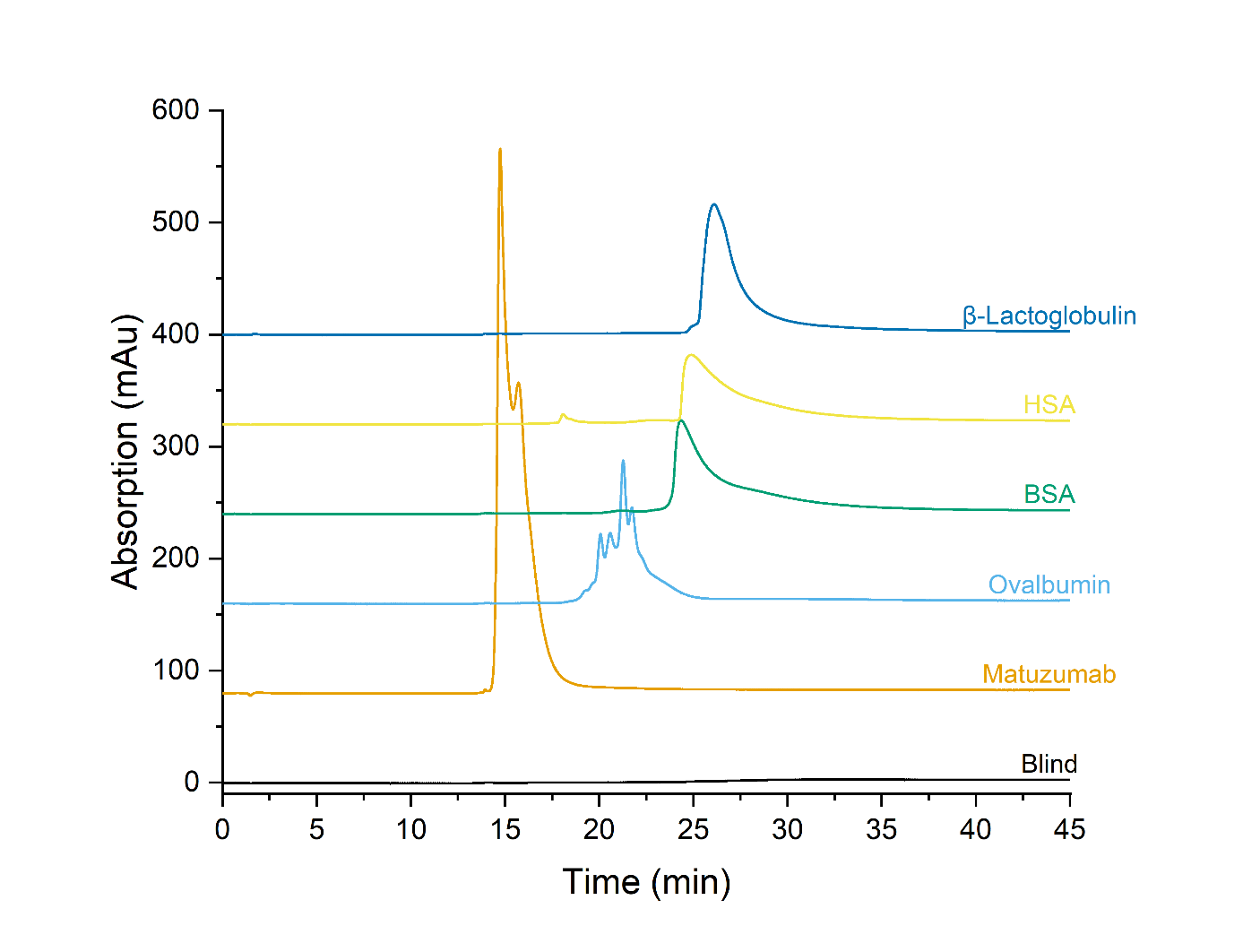


**SI Fig. 7** Exemplary chromatograms (280 nm) of Matuzumab, Ovalbumin, BSA, HSA and β-lactoglobulin with a protein concentration of 1 mg/mL in MPA and an injection volume of 500 µL; each trace with 80 mAu offset.


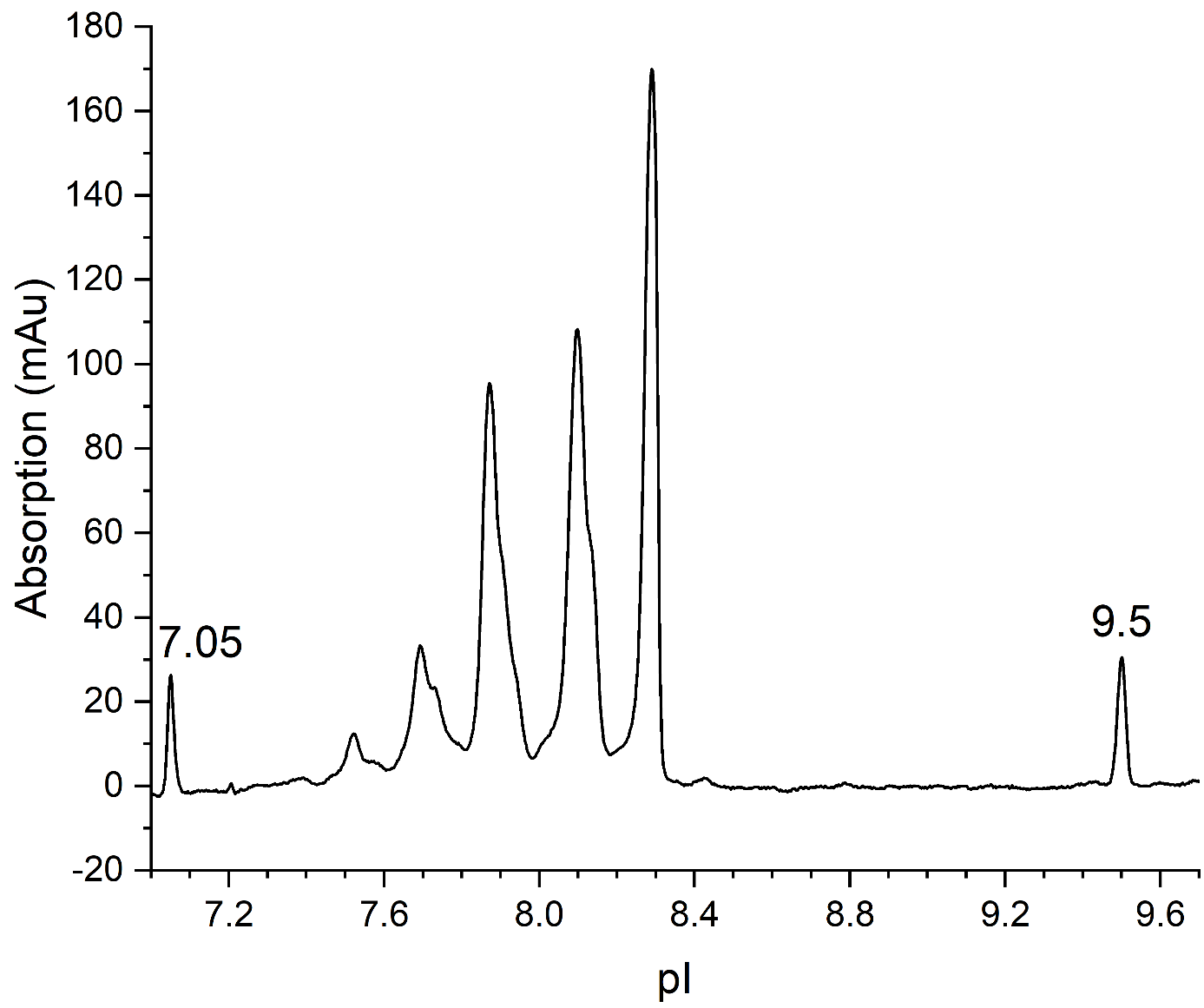


**SI Fig. 8** Imaged capillary isoelectric focusing electropherogram of Matuzumab. The leftmost peak is pI marker 7.05 and the rightmost peak is pI marker 9.5. In between the pI markers, Matuzumab presents a charge profile with distinct peaks at pI 7.5, 7.7, 7.9, 8.1 and 8.3 (main peak).
Experimental conditions: Maurice C., controlled with Compass for iCE version 2.1.0 (also used for data evaluation), was provided by ProteinSimple (San Jose, USA). Ready-to-use icIEF cartridges equipped with a fluorocarbon capillary (100 µm inner diameter, 5 cm effective length), 1% methyl cellulose (MC) solution, anolyte (80 mM phosphoric acid in 0.1% MC), catholyte (100 mM sodium hydroxide in 0.1% MC), pI marker 7.05, pI marker 9.5, urea and L-arginine were all from ProteinSimple (San Jose, USA). Pharmalyte® (PL) 8 – 10.5 and PL 5 – 8 were purchased from GE Healthcare (Uppsala, Sweden). For analysis, 80 µL of a master mix consisting of 19 µL water, 84 µL 1% MC, 72 µL 10 M urea, 2 µL 500 mM L-arginine, 7 µL PL 8 – 10.5, 2 µL PL 5 – 8, 2 µL pI marker 7.05 (1 mg/mL in water) and 2 µL pI marker 9.5 (1 mg/mL in water), was admixed with 20 µL of Matuzumab (1 mg/mL in water). The sample was vortexed, centrifuged (10 000 × g/3 min) and 80 µL transferred into a 96-well plate in the autosampler (temperature 10 °C). Separation parameters were: loading time 55 s; focusing 1 min at 1500 V, followed by 13 min at 3000 V; detection through UV absorbance at 280 nm.
